# Differential expression of tetraspanin superfamily members in dendritic cell subsets

**DOI:** 10.1371/journal.pone.0184317

**Published:** 2017-09-07

**Authors:** Malou Zuidscherwoude, Kuntal Worah, Alie van der Schaaf, Sonja I. Buschow, Annemiek B. van Spriel

**Affiliations:** 1 Department of Tumor Immunology, Radboud Institute for Molecular Life Sciences, Radboud University Medical Center, Nijmegen, The Netherlands; 2 Centre for Molecular and Biomolecular Informatics (CMBI), Radboud University Medical Center, Nijmegen, the Netherlands; 3 Department of Gastroenterology and Hepatology, Erasmus MC-University Medical Center, Rotterdam, the Netherlands; Universitatsklinikum Wurzburg, GERMANY

## Abstract

Dendritic cells (DCs), which are essential for initiating immune responses, are comprised of different subsets. Tetraspanins organize dendritic cell membranes by facilitating protein-protein interactions within the so called tetraspanin web. In this study we analyzed expression of the complete tetraspanin superfamily in primary murine (CD4+, CD8+, pDC) and human DC subsets (CD1c+, CD141+, pDC) at the transcriptome and proteome level. Different RNA and protein expression profiles for the tetraspanin genes across human and murine DC subsets were identified. Although RNA expression levels of CD37 and CD82 were not significantly different between human DC subsets, CD9 RNA was highly expressed in pDCs, while CD9 protein expression was lower. This indicates that relative RNA and protein expression levels are not always in agreement. Both murine CD8α+ DCs and its regarded human counterpart, CD141+ DCs, displayed relatively high protein levels of CD81. CD53 protein was highly expressed on human pDCs in contrast to the relatively low protein expression of most other tetraspanins. This study demonstrates that tetraspanins are differentially expressed by human and murine DC subsets which provides a valuable resource that will aid the understanding of tetraspanin function in DC biology.

## Introduction

Dendritic cells (DCs) are highly specialized immune cells that can sense tumor and microbial antigens and initiate both cellular and humoral immune responses. The complexity of the DC network has expanded enormously in the last decade by the identification of multiple different DC subsets. These subsets have been characterized by ontogeny, anatomical location, phenotypical markers, gene expression programs, and functionality [1,2]. The remarkable heterogeneity in DC subtypes may underlie a broad variety in the type, strength and duration of immune responses that may lead to either immunity or tolerance.

The DC network is largely conserved between mouse and human, although subset-discriminatory (cell surface) markers are different between the two species. In human peripheral blood, DC subsets are classified into plasmacytoid DCs (pDCs, BDCA4+) and two myeloid DC subsets: CD141+ (BDCA3+) and CD1c+ (BDCA1+) cells, also referred to as classical DC1 (cDC1) and cDC2, respectively [3]. These DC subsets are not only present in blood but have also been detected in different human lymphoid and non-lymphoid organs. In contrast to humans, for practical reasons murine DCs have mostly been studied in lymphoid organs such as the spleen, rather than in blood, and include plasmacytoid DCs (pDCs, CD11c int, B220+) and two myeloid DC subsets: CD8α+ (CD11b- CD11c+) DCs and CD4+ (CD11b+ CD11c+) DCs [4,5]. Although there are both phenotypical and anatomical differences between murine and human DC subsets, they share many functional properties [6]. Both human and murine pDCs have the capacity to produce vast amounts of type I interferons (IFNα and IFNβ) and as such are important in the induction of antiviral immune responses. The cDC1 subsets (human CD141+ and murine CD8α+ DCs) share the ability to mediate efficient antigen cross-presentation leading to activation of CD8+ T cells, whereas the cDC2 subsets are more efficient in stimulating CD4+ T cell responses and polarization towards Th2 and Th17 responses [2].

DCs interact with their environment (i.e. tissue surroundings, pathogens/tumor cells and other immune cells) through immunoreceptors that are embedded in the plasma membrane. It is well-established that these immunoreceptors (including major histocompatibility complex (MHC) molecules, pattern-recognition receptors (PRRs) and adhesion proteins) are non-randomly distributed at the cell surface and organized in domains. This organization not only increases receptor avidity, but also allows for receptor cross-talk and spatial regulation of receptor signaling. For example, MHC-peptide complexes on DCs are pre-clustered, permitting simultaneous engagement of multiple T cell receptors (TCRs) to overcome the required signaling threshold for T cell activation [7–10]. Clustering of immunoreceptors in DCs occurs in several specialized membrane domains, including caveolae, lipid rafts, and tetraspanin-enriched microdomains that each have a distinct organization and function [11–16]. In this study, we focused on the organizers of the latter domain, the tetraspanins, which are widely expressed but so far have been little studied in DC subsets.

Tetraspanins belong to the superfamily of four-transmembrane proteins that share a CCG motif and conserved cysteine residues in the large extracellular loop [17,18]. They are expressed on the plasma membrane as well as on intracellular membranes. Tetraspanins are not classical ligand-binding receptors that bind ligands *in trans*, instead they mostly interact with other proteins *in cis*. Documented binding partners include different immunoreceptors (MHC, CD4, CD8, C-type lectin receptors, and others), integrins and signaling proteins, and tetraspanins regulate their lateral organization [14,19]. It has been estimated that DCs express approximately 20 tetraspanins of the 33 different family members that have been identified in mammalian cells [20,21]. Despite the essential role of cell surface receptors for DC function, studies on the role of tetraspanin proteins in regulating DC receptors have only recently been initiated. We and others have shown that DC migration is regulated by tetraspanins CD37 and CD81. CD81 was found to be required for the formation of membrane protrusions during adhesion-dependent DC migration, and CD37-deficiency leads to impaired DC migration from skin to lymph nodes in mice [22,23]. In contrast, the predominantly intracellularly expressed tetraspanin CD63 may slow down DC migration via a mechanism that is yet unclear [24]. Other studies have reported on the involvement of tetraspanins (CD37, CD151, CD9, CD81, CD82) in antigen presentation by DCs by regulating MHC interactions, the formation of the immune synapse, or through exosomes [25–29].

Together these studies underscore that tetraspanins play an important role in regulating DC surface receptor function. The different DC subsets differ greatly in the set of surface receptors they express [30], and consequently may require a distinct set of tetraspanins for receptor regulation. Moreover, variation in expression of tetraspanins between DC subsets could equip DCs with cell specific functions by differential regulation of the same cell surface receptor in one DC subset with respect to other subsets. Here, we directly compared the expression of the complete tetraspanin superfamily in primary murine and human DC subsets at the RNA and protein level and discuss potential implications for DC biology.

## Materials and methods

### Microarray data

For RNA analysis, publically available affymetrix CEL files containing expression data of resting blood derived human pDCs, CD1c+ and BDCA3 (CD141+) mDCs were downloaded from ArrayExpress (accession: E-TABM-34). The FACS sorting and subsequent sample preparation and analysis is described in the associated study by Lindstedt *et al*. [31]. For mouse DCs, expression data generated as part of the Immunological Genome Project (Immgen) of resting spleen derived-pDCs (GSM605840-GSM605842), CD8α DCs (GSM538258-GSM538260) and CD4+ DCs (GSM538248-GSM538250) was used (GSE 15907) [32]. Data were downloaded from the Gene Expression Omnibus. Sample preparation, gating and analysis is described in detail on the Immgen website (http://www.immgen.org).

The raw files were processed in the R programming environment and intensity values were normalized using the RMA normalization function of the affy package [33]. The normalization was evaluated by boxplots (S1 Fig). Specific annotation packages for human (hgu133plus2.db) and mouse (mogene10sttranscriptcluster.db) were used to map array probe identifiers to corresponding species specific Gene Symbols. When multiple Probesets target the same gene, only one ProbeSet was included in the final dataset. In these cases, the ProbeSet with the highest summed intensity across all samples was selected. From the curated tetraspanin genes sets (http://www.genenames.org/genefamilies/TSPAN) we were able to retrieve 32 tetraspanins in the human dataset and 31 tetraspanins in the mouse dataset. For genes with an expression level below 5 we did not find any ANOVA significant differences. Because of the increasing influence of background noise these data were considered less reliable. Therefore genes with expression levels below 5 in 2-out-of-3 donors in all DC subsets (6 genes in the human set and 2 genes in the mouse set) were not presented in the main results and are only included in S1 Table, which provides the probe identifiers, RNA expression levels and statistical analyses of all members of the tetraspanin family.

ANOVA was applied on the ^2^log-transformed values of the retrieved tetraspanins for human and mouse data separately. p-values were corrected for multiple testing using the Benjamini & Hochberg correction. Genes with a resulting p-value <0.05 were considered as differentially expressed and subjected to further post hoc t-testing. For the generation of heat maps, human and murine datasets were separately z-scored (setting the data to a mean = 0 and a variance = 1), and heat maps were generated using freely availably GeneE program (http://www.broadinstitute.org/).

### Cells

Human blood peripheral blood mononuclear cells (PBMCs) were isolated from buffy coats by Ficoll density centrifugation (Lucron Bioproducts). Buffy coats of healthy individuals (Sanquin) were obtained after written informed consent and according to institutional guidelines and the Helsinki declaration. This was verified by the local institutional review board (Commissie mensgebonden onderzoek (CMO)). Mice of the C57Bl/6j background were bred in the Central Animal Laboratory of the Radboud university medical center, and sacrificed by cervical dislocation or exposure to rising concentrations of CO_2_. Single cell suspensions of murine spleens were made by DNAse and collagenase treatment, after which DCs were enriched by Nycodenz density centrifugation as described in [34]. All murine studies complied with European legislation (directive 2010/63/EU of the European Commission) and these studies were approved by local authorities (CCD, The Hague, the Netherlands) for the care and use of animals with related codes of practice.

### Flow cytometry

Antibodies used in this study are listed in S3 Table. Human PBMCs were stained with viability dye e780 in phosphate buffered saline (PBS) before fixation with 2% paraformaldehyde (PFA) in PBS. Murine cells were fixed with 4% PFA. Cells were permeabilized with 0.5% saponin (Sigma) in PBS with 1% BSA and 0.05% NaN_3_ (PBA). Cells were blocked and stained in 1% human serum (human cells) or 2% goat serum (murine cells), 0.5% saponin in PBA. First, cells were stained with anti-tetraspanin antibodies or isotype controls, followed by labelled secondary antibodies. Next, free arms of bound secondary antibodies were blocked with mouse or rabbit serum, and subsequently stained with antibodies for DC subset identification. The gating strategy for identification of DC subsets was similar to the gating strategy used for the cell isolation for RNA analysis [31,32]. Human PBMCs were analyzed on the FACSVerse (BD Biosciences) and murine cells on the FACSCalibur (BD Biosciences). Data analysis was done using FlowJo software (version 9.7, TreeStar Inc.). For statistical analysis of flow cytometry data the mean fluorescent intensities (MFI) of isotype control stainings were first subtracted from the specific stainings. In some cases this resulted in negative values. To allow log transformation for further statistical analysis all values were then increased with the lowest possible, for each protein different, set value that would render all values for that protein positive and resulting values were subsequently 2log transformed. Three-group ANOVA was then applied on the 2log-transformed mean fluorescence intensity values for human and mouse datasets separately. ANOVA p-values were corrected for multiple testing using the Benjamini & Hochberg procedure. Genes with a corrected p-value <0.05 were considered as differentially expressed and subjected to further post hoc t-testing.

## Results

### Tetraspanins are differentially expressed by human DC subsets

RNA expression data of tetraspanins in human DCs subsets was retrieved from a publically available dataset [31], which provides relative mRNA expression levels of tetraspanin superfamily members in CD1c+ DCs, CD141+ DCs and pDCs derived from the blood of 3 healthy donors. From these files we were able to identify 32 different tetraspanins expressed by human DCs of which 6 were excluded from the analyses due to low expression levels (< 5 in 2-out-of-3 donors) which prevented reliable assessment of differential expression between subsets. Information on these tetraspanins that were hardly detectable is included in S1 Table.

**Fig 1:**
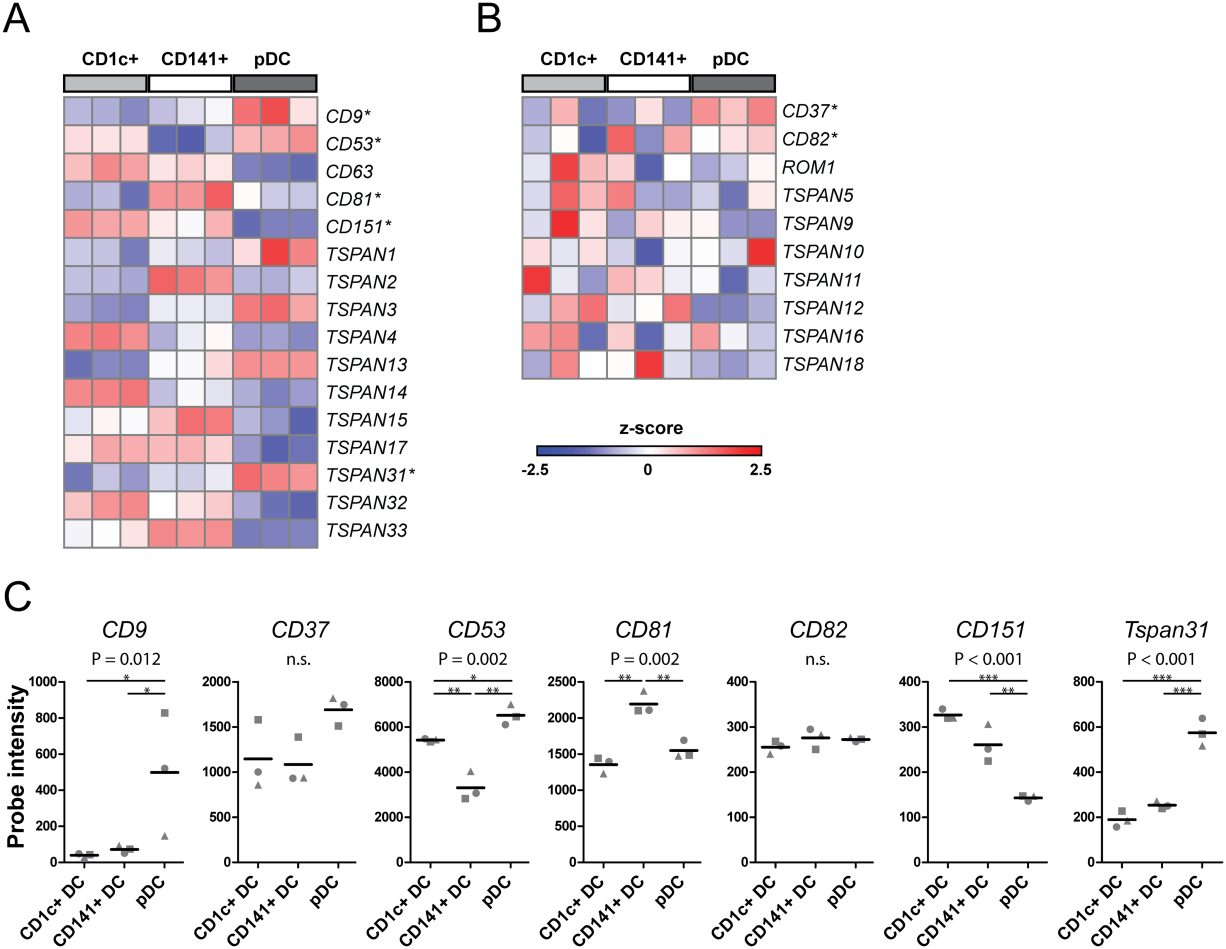
Relative mRNA expression levels of tetraspanins in human DC subsets. Z-scored values of (A) differentially expressed genes (ANOVA p<0.05) and (B) non-differentially expressed genes (ANOVA p>0.05 and expression >2^5^ in at least two donors in one DC subset). Asterisks mark genes that were selected for further analysis. See S1 Table for selected probes and statistics on expression data of all members of the tetraspanin family. (C) Gene expression levels of tetraspanins in DC subsets, normalized probe intensities are plotted, each symbol represents one healthy donor. P-values displayed above each plot represent the result of ANOVA to which multiple testing correction was been applied (n.s. = non-significant). * p<0.05, ** p< 0.01 ***p<0.001 by post-hoc t-testing. See also S1 Table for full results of the statistical analysis.

From the resulting 26 different human tetraspanins, 16 appeared differentially transcribed between the DC subsets (Fig 1A; S1 Table). The expression profiles differed greatly between the tetraspanin genes: *CD9*, *CD53*, *TSPAN1*, *TSPAN3*, *TSPAN13* and *TSPAN31* were relatively high expressed in pDCs compared to CD1c+ DCs and CD141+ DCs, whereas the other tetraspanin genes displayed higher mRNA levels in mDC subsets compared to pDCs (*CD63*, *CD151*, *TSPAN17*). In addition, several tetraspanin genes were differentially transcribed between mDC subsets CD1c+ and CD141+ DCs (e.g. *CD53*, *CD81*, *TSPAN2*, *TSPAN4*, *TSPAN14)*. For the tetraspanin genes depicted in Fig 1B the expression pattern between DC subsets was much less consistent and concordantly no significant differences in their mRNA expression levels between the different DC subsets were found. Based on the microarray data, our specific interest and the availability of antibodies, we selected 7 tetraspanin genes for in-depth analysis. Plotting of the untransformed RNA expression levels of these tetraspanins highlighted that although *CD9* was remarkably high expressed in pDCs compared to the other subsets, differences in expression levels between the DC subsets were generally small, but consistent between the different donors (*CD53*, *CD81*, *CD151* and *TSPAN31)*. mRNA expression levels of *CD37* and *CD82* were not significantly different between DC subsets, but are of interest for further analysis because of their potential roles in antigen presentation and migration that are key to DC function [22,23] (Fig 1C).

**Fig 2:**
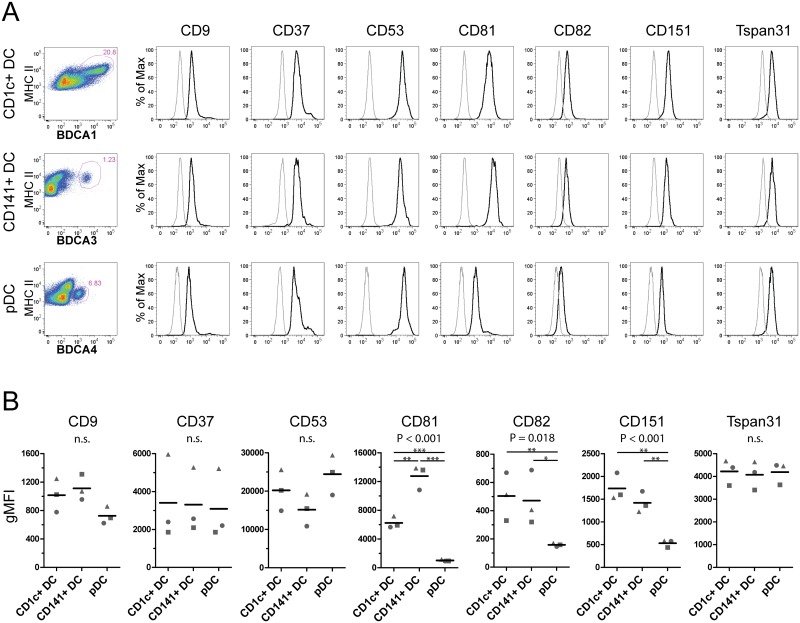
Protein expression of tetraspanins on human DC subsets. (A) Flow cytometric analysis of total tetraspanin protein expression on fixed permeabilized DC subsets. Far left: dot plots of viable Lin-MHC II+ cells. CD1c+ DCs were identified as BDCA1+, CD141+ DCs as BDCA3+, and pDCs as BDCA4+ cells. See S2 Fig for full gating strategy. Histograms of tetraspanin expression (black curve) and isotype control staining (grey curve) on gated cells depicted in the dot plots. (B) Tetraspanin expression of 3 healthy donors, geometric mean fluorescence intensity (gMFI) normalized for isotype control binding, each symbol represents one donor. P-values displayed above each plot represent the result of ANOVA to which multiple testing correction was been applied (n.s. = non-significant). * p<0.05, ** p< 0.01 ***p<0.001 by post-hoc t-testing. See also S2 Table for full results of the statistical analysis.

Next, for the selected set of 7 tetraspanins on human DC subsets, we investigated whether RNA expression values were indicative of protein expression levels. Total protein expression levels were measured on CD1c+ DCs, CD141+ DCs and pDCs by flow cytometry. Gating strategies for the identification of these DC subsets were matched to those used for DC subset cell isolation in the RNA dataset [31]. For most tetraspanins, the observed differential expression between the DC subsets at the mRNA level was confirmed at the protein level (Fig 2A; Table 1). The protein data, however, was not always in agreement with the RNA expression data. We observed a lower CD9 and CD82 protein expression on pDCs compared to CD1c+ or CD141+ DCs and equal levels of TSPAN31 (Fig 2B), which was in contrast to their relative mRNA expression levels in pDCs. These results indicate that CD9, CD82 and TSPAN31 protein expression in pDCs is possibly modulated by decreased mRNA stabilisation and/or increased protein degradation. For the other tetraspanins studied, mRNA and protein levels were largely in agreement (Table 1). Although CD37 protein expression varied greatly between donors, similar expression levels were found between the DC subsets of each donor. CD53 was highly expressed in pDCs which was in sharp contrast to the relatively low protein expression levels of most other tetraspanins on pDCs (CD9, CD81, CD82 and CD151). CD81 was markedly higher expressed on CD141+ DCs compared to the other DC subsets, and CD151 protein expression was highest on CD1c+ DCs closely followed by CD141+DCs and much lower on pDCs.

**Table 1:** Tetraspanin protein expression on DC subsets.

	Human		Mouse	
**CD9**	D	pDC low	A	CD8α+ DC high, pDC very low
**CD37**	A	Similar expression		N.A.
**CD53**	A	pDC high	D	pDC low
**CD81**	A	CD141+ DC high, pDC very low	A	CD8α+ DC high, pDC very low
**CD82**	D	pDC low		N.A.
**CD151**	A	pDC low	D	CD4+ DC low, CD8α high
**Tspan31**	D	Similar expression		N.A.

D: RNA and protein expression profile disagree. A: RNA and protein expression profile agree. N.A.: not analyzed. Remarks on the relative protein expression profile of each tetraspanin are described.

### Tetraspanins are differentially expressed by murine DC subsets

**Fig 3:**
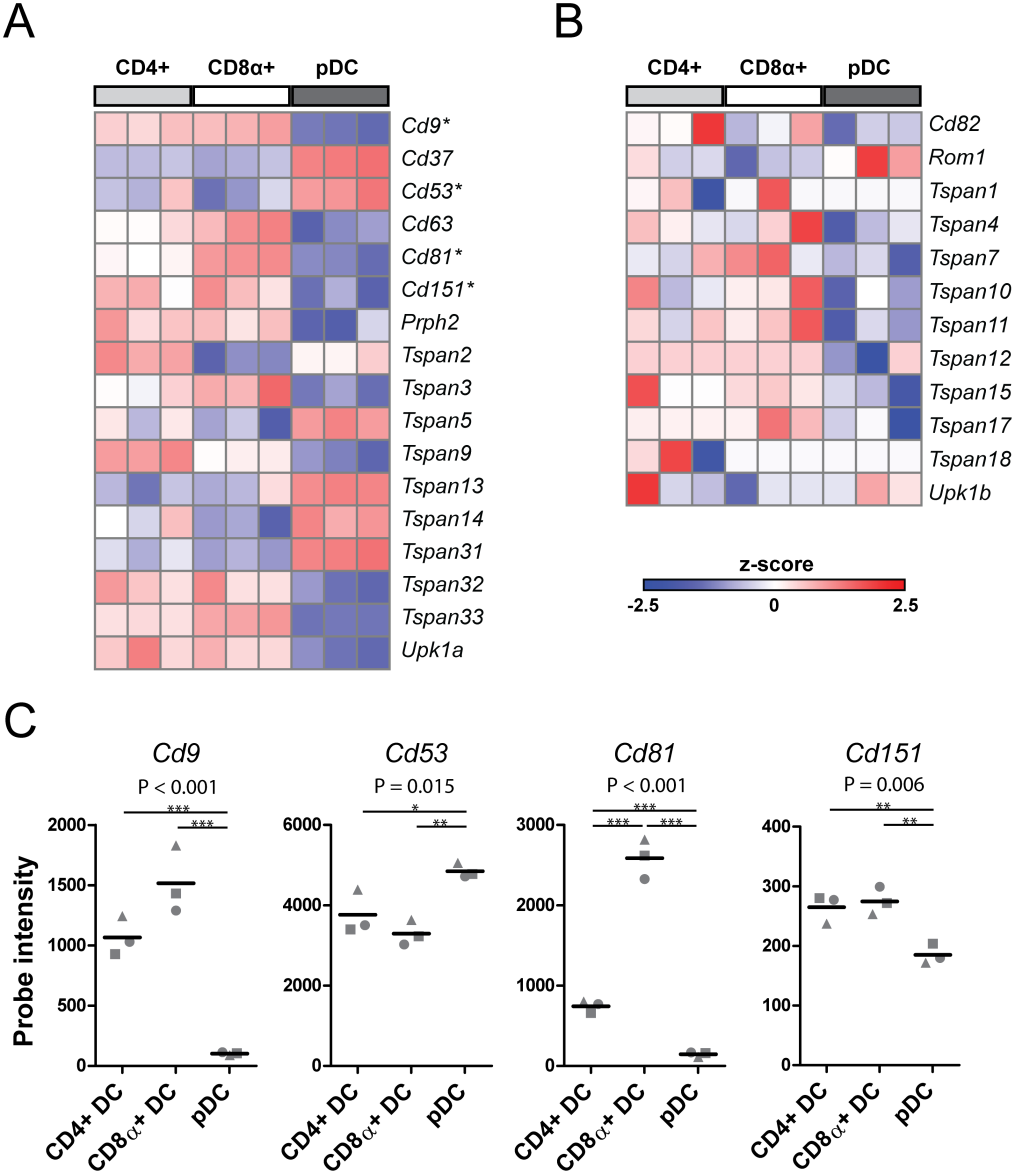
Relative mRNA expression levels of tetraspanins in murine DC subsets. Z-scored values of (A) differentially expressed genes (ANOVA p<0.05) and (B) non-differentially expressed genes (ANOVA p>0.05 and expression > 2^5^ in at least two mice in one subset). Asterisks mark genes that were selected for further analysis. See S1 Table for selected probes and statistics on expression data of all members of the tetraspanin family. (C) Gene expression levels of tetraspanins in DC subsets, probe intensities are plotted, each symbol represents one mouse. P-values displayed above each plot represent the result of ANOVA to which multiple testing correction was been applied (n.s. = non-significant). * p<0.05, ** p< 0.01 ***p<0.001 by post-hoc t-testing. See also S1 Table for full results of the statistical analysis.

To investigate the conservation of tetraspanin expression in the murine DC-subset equivalents, we retrieved mRNA expression data of tetraspanins for spleen-derived CD11b+CD4+DCs, CD11b-CD8α+ DCs and pDCs from the public ImmGen repository [32]. Within the DC subset data containing mRNA expression from 3 mice 31 tetraspanin genes were identified, of which 29 different tetraspanin genes met the selection criteria to be analyzed further. 17 tetraspanin genes were found to be differentially transcribed between the different DC subsets (Fig 3A; S1 Table). Similar to their human counterparts, these murine tetraspanin genes showed different expression profiles across the DC subsets. *Cd37*, *Cd53*, *Tspan5*, *Tspan13*, *Tspan14* and *Tspan31* were relatively high expressed in pDCs, whereas many other tetraspanin genes demonstrated higher mRNA levels in mDCs compared to pDCs (Fig 3A). Interestingly, differences in relative tetraspanin expression between CD4+ and CD8α+ DCs seemed less pronounced than the differences observed between the human mDC counterparts. The tetraspanin genes depicted in Fig 3B were not found to be differentially expressed between the DC subsets. In contrast to the human RNA profile, but in line with the human protein data, *Cd9* was expressed at relatively low levels on pDCs compared to CD4+ and CD8α+ DCs (Fig 3C). *Cd53* was expressed at relatively high levels on murine pDCs and *Cd81* on CD8α+ DCs, which matched the human *CD53* and *CD81* expression profiles.

**Fig 4:**
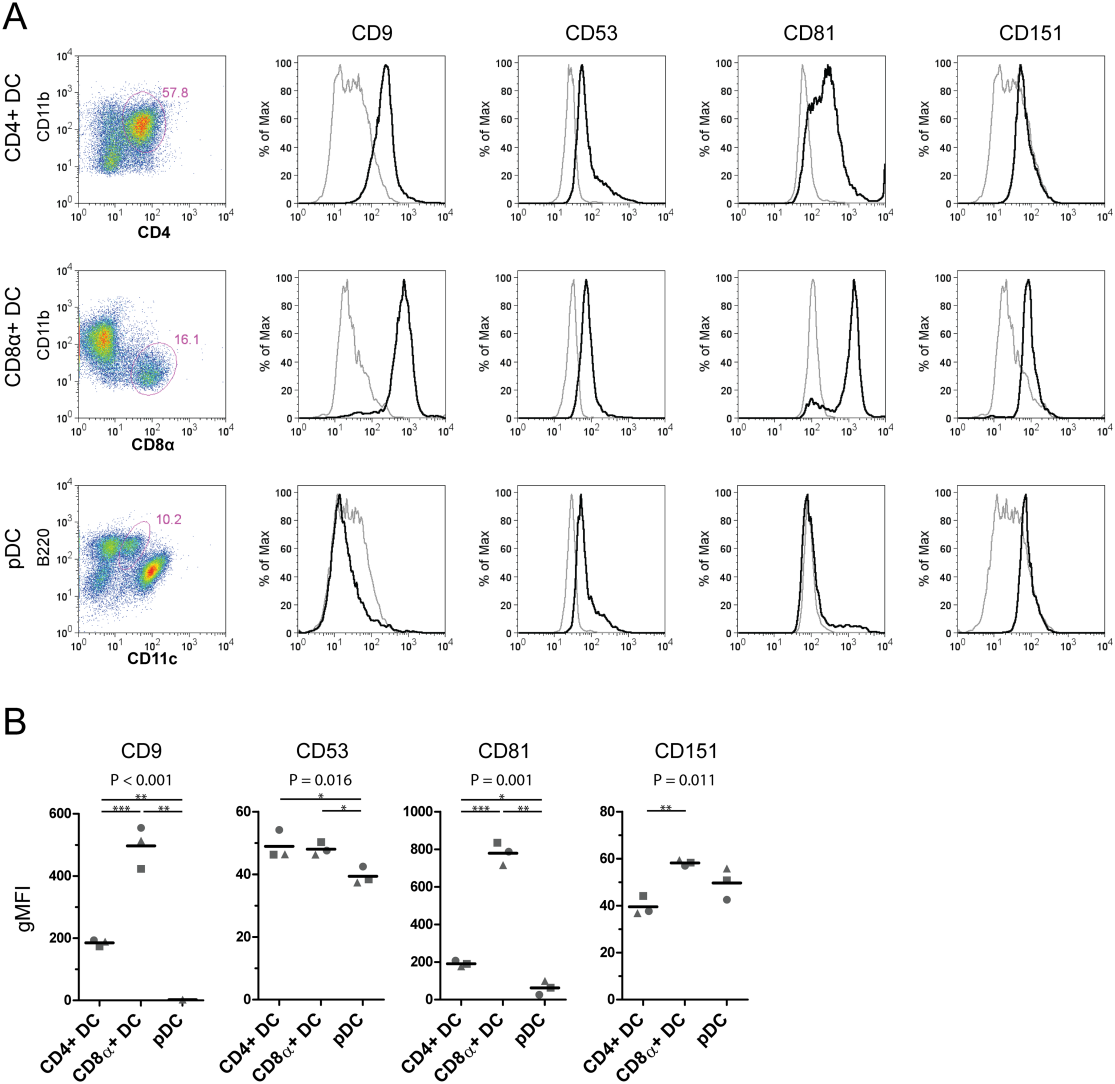
Protein expression of tetraspanins on murine DC subsets. (A) Flow cytometric analysis of total tetraspanin protein expression on DC subsets. Cells from spleen were enriched for DCs as described in the materials and methods. Far left: dot plots of viable CD11c+ cells (CD4+ and CD8α+ DCs) or viable pDCs. CD4+ DCs were identified as CD4+CD11b+, CD8α+ DCs as CD8α+CD11b-, and pDCs as B220+CD11c^int^ cells. See S3 Fig for full gating strategy. Histograms of tetraspanin protein expression on gated cells depicted in the dot plots (black line) and isotype controls (grey line). (B) Average tetraspanin expression of 3 individual mice, geometric mean fluorescence intensity (gMFI) normalized for isotype control binding, each symbol represents one mouse. P-values displayed above each plot represent the result of ANOVA to which multiple testing correction was been applied (n.s. = non-significant). * p<0.05, ** p< 0.01 ***p<0.001 by post-hoc t-testing. See also S2 Table for full results of the statistical analysis.

Subsequently we measured the protein expression levels of selected tetraspanins for the murine DC subsets. Gating strategies for the identification of CD11b+CD4+ DCs, CD11b-CD8α+ DCs and pDCs were matched to those used for DC subset cell isolation in the RNA dataset [32]. Due to limited availability of antibodies recognizing mouse tetraspanins the analysis was restricted to total protein expression of CD9, CD53, CD81 and CD151 (Fig 4A). Notably, CD9 and CD81 were absent on pDCs which is line with the human data (Fig 2). In contrast, CD8α+ DCs displayed relatively high protein levels of these tetraspanins (Fig 4B), which was again similar to our observations in human CD141+ mDCs. On the other hand, the expression pattern of CD53 differed greatly between murine mRNA and protein data as well as between murine and human protein data (Table 1). Opposite to the relatively high *Cd53* mRNA levels, lower protein levels of CD53 were observed on murine pDCs, compared to CD4+ and CD8α+ DCs. This indicated that also CD53 protein expression levels are not solely regulated by the amount of mRNA. Similarly, CD151 protein expression on murine pDCs was in the same range as CD151 expression on the other DC subsets, in contrast to the low *Cd151* expression levels on pDCs measured at the mRNA level.

## Discussion

Here, a complete overview of expression of members of the tetraspanin superfamily is provided in human and murine DC subsets at both the mRNA and protein level. We demonstrate that many tetraspanins are differentially expressed between cDC1s, cDC2s and pDCs, indicating they may have a role in sustaining subset identity or functional differences. Moreover, different expression profiles were found between individual tetraspanin genes, which may reflect the reported non-redundant functions of members of the tetraspanin superfamily [14,19,35].

We are aware of the limitations of our study (e.g. small sample size, variation between donors), and as a result we did not obtain clear results in all cases (human CD9, CD53). Despite these limitations however, consistent tetraspanin protein expression patterns between DC subsets throughout the donors tested were identified for several tetrapanins (human CD81, CD82, CD151; Murine CD9, CD53, CD81, CD151) while for others our results consistently indicate that no large differences may exist (human CD37, CD53, Tspan31) (Figs 1A and 3A). These results represent a good starting point for further tetraspanin research on DC subsets.

Although for most tetraspanins RNA and protein data were in agreement we identified also several cases where protein expression and RNA expression were not consistent (human CD9, CD82, Tspan31; murine CD53, CD151; Table 1). Because we used publically available microarray data this could be caused by the fact that we did not use data from the exact same cells, despite the well-matched gating strategy. Our recent observation that the correlation between RNA and protein expression across different isolates of the same cell types from different individuals is very well preserved [36] however, argues that this may not be the primary explanation for this discrepancy. Possibly, posttranscriptional regulatory mechanisms are involved in regulating RNA translation and protein stability for these tetraspanins.

Although DC subsets share their function as professional antigen-presenting cells, they are highly specialized when it comes to pathogen recognition, antigen uptake and processing, T cell co-stimulation, cytokine secretion and migratory routes. All these processes are regulated by transmembrane proteins and downstream intracellular signaling cascades. After antigen uptake, DCs migrate towards lymph nodes to activate naïve T lymphocytes. Within the lymph node DCs present non-self peptides to CD4+ T cells on MHC class II, or to CD8+ T cells on MHC class I molecules. The interaction between MHC class II and tetraspanins (CD9, CD37, CD53, CD63, CD81 and CD82) is well documented [15]. It has been suggested that tetraspanins regulate both plasma membrane stability and intracellular trafficking and loading of MHC molecules [37,38]. The relative low expression of tetraspanins (CD9, CD81, CD82) on pDCs may reflect a regulatory role for these tetraspanins in processes that are important in cDC function, such as phagocytosis [39], antigen processing [40] or MHC class II trafficking [41].

Although CD141+ DCs are able to present antigen via MHC class II to CD4+ T cells, they stand out in their ability to efficiently cross-present antigens from dying or virus infected cells via MHC class I to CD8+ T cells. CD53, CD81 and CD82 have been reported to interact with MHC class I [42], and it will be interesting to study the role of these tetraspanins in antigen cross-presentation or receptor mediated uptake of remnants of dying cells by CD141+ DCs and CD8α+ DCs. CD81 is of particular interest, since we observed CD81 to be highly expressed in both human CD141+ DCs and murine CD8a+ DCs.

Notable was the relative high protein expression of tetraspanin CD53 on all human DC subsets and in particular on human pDCs. This result is in line with our recent comparative proteome analysis of human DC subsets that indicated higher CD53 expression on pDCs [36]. CD53 is exclusively expressed on cells of the hematopoietic system and highly expressed on antigen-presenting cells [43]. pDCs differ from the other DC subsets by their unique expression of virus recognizing PRRs, their ability to secrete high amounts of type I interferon, and their unique migratory and adhesive properties. We observed that pDCs expressed relatively low protein levels of many tetraspanins, even though mRNA levels were high (CD9, CD81, CD82). It is possible that pDCs, more than the other DC subsets, regulate tetraspanin protein expression via additional mechanisms like posttranslational modifications, protein and/or RNA degradation or microRNAs. In colorectal carcinoma, *Tspan1* mRNA is targeted by microRNA-638 suggesting that some tetraspanins are prone to this latter regulatory mechanism [44]. Moreover, tetraspanin expression levels have been reported to change upon activation and differentiation of other immune cells [45], thus it would be valuable to investigate differential expression of tetraspanins in DCs during differentiation and activation. This is illustrated by a recent study on CD37 and CD82 expression and function on immature and mature murine bone marrow derived DCs. CD37, which promotes migration but restrains antigen presentation, is down-regulated upon DC maturation, whereas CD82, which restrains migration but promotes antigen presentation, is up-regulated in mature DCs [46].

The expression pattern of tetraspanins in DC subsets was not always conserved between human and mouse, as shown here for CD53 and CD151. This may be due to the different anatomical location the subsets were obtained from (i.e. blood versus spleen), and the resulting differential need for integrin activity. Tetraspanins are known to control integrin avidity and its downstream signaling events [22,47]. On B cells, CD37 controls integrin α4β1 clustering and function, leading to defective AKT kinase signaling and increased apoptosis of B cells in the absence of CD37 [48]. Moreover, CD37-deficient dermal DCs show impaired directional migration to draining lymph nodes, which contributes to the poor cellular immune responses observed in *Cd37-/-* mice [23]. CD151 strongly interacts with and modulates the function of laminin-binding integrins, thereby affecting cell adhesion and migration [49,50]. This is apparent in *Cd151-/-* mice that are impaired in wound healing [51], and are protected against tumor cell metastasis [52]. It would be interesting to investigate whether differential expression of CD151 in DC subsets influences integrin-dependent DC adhesion and migration.

Due to their interactions with various immune receptors and adhesion molecules, tetraspanins play important and diverse roles in many immunological processes including antigen presentation, cell adherence and cytokine production. This study on tetraspanin expression on the different DC subsets, combined with increased understanding into specific functions of DC subsets provides a valuable resource to elucidate which biological processes are regulated by individual tetraspanins.

## Supporting information

S1 Table RNA data file. (PDF)

S2 Table Protein data file. (PDF)

S3 Table Antibodies. (DOCX)

S1 Fig Data normalization. Box plots of normalized probe intensity distributions. Complete data set of expression values of human blood DC subsets (A) and murine spleen DC subsets (B) were normalized using the RMA normalization function and ^2^log transformation.
(TIF)

S2 Fig Gating strategy human DC subsets. PBMCs were stained with specific antibodies and analyzed by flow cytometry. (A) Cell debris (left) and cells positive for dead cell marker were excluded (middle). DCs positive for MHC class II and negative for lineage markers CD3, CD14, CD16, CD19, CD20 and CD56 were selected (right). (B) CD1c+ DCs were gated on MHC class II and BDCA1 positivity (left). Cells positive for BDCA3 and BDCA4 were excluded (right) from tetraspanin expression analysis. (C) CD141+ DCs were gated on MHC class II and BDCA3 positivity (left). Cells positive for BDCA1 and BDCA4 were excluded (right) from tetraspanin expression analysis. (D) pDCs were gated on MHC class II and BDCA4 positivity (left). Cells positive for BDCA1 and BDCA3 were excluded (right) from tetraspanin expression analysis.
(TIF)

S3 Fig Gating strategy murine DC subsets. Splenic cells were enriched for DCs, stained with specific antibodies and analyzed by flow cytometry. Dead cells were excluded based on forward and side scatter characteristics. Upper: CD11c+ cells were gated on CD11b+ CD4+ to determine tetraspanin expression on CD4+ DCs. Middle: CD11c+ CD11b- CD8α+ cells were selected for tetraspanin expression analyses. Lower, pDCs: B220+ CD11c^int.^ cells were gated on CD8α+ for tetraspanin expression analyses.
(TIF)
