# Supplementary material for: Differential expression of tetraspanin superfamily members in dendritic cell subsets
Source: PLoS One. 2017 Sep 7;12(9):e0184317. doi: 10.1371/journal.pone.0184317 (PMC5589240; doi:10.1371/journal.pone.0184317)
Supplement: S1 Table — (PDF) [file pone.0184317.s001.pdf]

Human ID			2log probe expression									
Probe Set ID	Gene Symbol	Entrez ID Human	Blood CD1c+R1	Blood CD1c+R2	Blood CD1c+R3	Blood CD141+ R1	Blood CD141+ R2	Blood CD141+ R3	Blood pDC R1	Blood pDC R2	Blood pDC R3	Anova P.value
201005_at	*CD9*	928	5.58	5.42	4.86	5.69	6.20	6.54	9.02	9.69	7.20	1.228E-02
204192_at	*CD37*	951	9.97	10.62	9.75	9.86	10.44	9.87	10.77	10.56	10.83	1.495E-01
203416_at	*CD53*	963	12.42	12.38	12.41	11.58	11.47	11.98	12.58	12.66	12.77	2.055E-03
200663_at	*CD63*	967	11.65	11.85	11.77	11.52	11.59	11.51	10.96	10.93	10.88	1.025E-04
200675_at	*CD81*	975	10.45	10.49	10.26	11.04	11.04	11.21	10.72	10.54	10.52	1.860E-03
203904_x_at	*CD82*	3732	8.01	8.07	7.91	8.20	7.97	8.14	8.06	8.09	8.11	3.719E-01
204306_s_at	*CD151*	977	8.41	8.32	8.32	7.97	7.81	8.26	7.08	7.20	7.20	4.684E-04
205806_at	*ROM1*	6094	6.16	6.55	6.33	6.29	5.93	6.22	6.06	6.12	6.23	3.467E-01
209114_at	*TSPAN1*	10103	5.81	5.79	5.49	5.96	5.86	5.77	6.22	6.94	6.61	1.644E-02
227236_at	*TSPAN2*	10100	4.70	4.63	4.27	8.29	7.94	7.48	4.57	4.45	4.86	1.025E-04
200972_at	*TSPAN3*	10099	10.04	9.86	9.78	10.61	10.61	10.52	11.86	12.00	11.49	1.633E-04
209263_x_at	*TSPAN4*	7106	8.25	8.32	8.16	6.80	7.21	7.42	6.67	6.70	6.53	5.339E-04
209890_at	*TSPAN5*	10098	5.17	5.52	5.36	5.47	5.08	5.07	5.15	4.99	5.27	4.558E-01
220968_s_at	*TSPAN9*	10867	6.10	6.45	6.19	6.03	6.21	6.17	6.17	6.01	6.00	3.467E-01
223795_at	*TSPAN10*	83882	7.17	7.11	7.17	7.09	6.95	7.13	7.13	7.10	7.36	3.467E-01
227610_at	*TSPAN11*	441631	5.71	5.15	4.97	5.39	5.32	5.17	5.20	4.85	5.12	5.113E-01
230625_s_at	*TSPAN12*	23554	5.18	5.34	5.40	5.20	5.24	5.39	5.09	5.09	5.14	1.169E-01
217979_at	*TSPAN13*	27075	6.45	7.11	6.99	10.31	10.24	11.60	13.75	13.71	13.50	1.025E-04
223314_at	*TSPAN14*	81619	8.17	8.20	8.27	7.10	7.42	7.30	7.00	6.75	6.91	1.633E-04
218693_at	*TSPAN15*	23555	4.42	4.59	4.50	4.77	5.09	5.04	4.24	4.12	3.90	2.550E-03
233236_at	*TSPAN16*	26526	7.25	7.27	6.97	7.19	6.96	7.11	7.25	7.12	7.07	7.828E-01
225235_at	*TSPAN17*	26262	8.04	8.29	8.26	8.21	8.24	8.12	7.56	7.29	7.40	8.549E-04
228819_at	*TSPAN18*	90139	5.30	5.44	5.36	5.36	5.50	5.33	5.30	5.29	5.32	3.467E-01
203227_s_at	*TSPAN31*	6302	7.29	7.83	7.52	7.97	7.90	8.08	9.32	9.15	9.01	4.684E-04
220558_x_at	*TSPAN32*	10077	8.61	9.21	9.34	7.88	8.22	8.53	6.83	6.18	5.91	1.209E-03
225775_at	*TSPAN33*	340348	9.80	9.98	10.41	11.76	11.57	11.68	8.13	8.18	8.23	5.503E-05
**Below Threshold**												
206625_at	*PRPH2*	5961	4.44	4.48	4.57	4.76	4.73	4.57	4.52	4.28	4.38	5.223E-02
209109_s_at	*TSPAN6*	7105	4.25	4.27	3.92	4.75	4.59	4.59	3.96	4.52	4.33	1.045E-01
202242_at	*TSPAN7*	7102	4.48	4.42	4.65	4.70	4.65	4.56	4.39	4.51	4.49	1.868E-01
203824_at	*TSPAN8*	7103	4.13	4.06	4.35	3.77	4.00	4.22	4.11	4.09	4.03	4.558E-01
214624_at	*UPK1A*	11045	4.35	4.40	4.40	4.42	4.37	4.36	4.33	4.60	4.32	8.946E-01
210064_s_at	*UPK1B*	7348	4.40	4.60	4.64	4.88	5.53	4.60	4.50	4.83	4.51	3.204E-01

Human ID			Z-Score								
Probe Set ID	Gene Symbol	Entrez ID Human	Blood CD1c+R1	Blood CD1c+R2	Blood CD1c+R3	Blood CD141+ R1	Blood CD141+ R2	Blood CD141+ R3	Blood pDC R1	Blood pDC R2	Blood pDC R3
201005_at	*CD9*	928	-0.67	-0.76	-1.10	-0.60	-0.29	-0.09	1.40	1.80	0.31
204192_at	*CD37*	951	-0.76	0.76	-1.28	-1.01	0.33	-0.99	1.10	0.61	1.23
203416_at	*CD53*	963	0.36	0.29	0.34	-1.43	-1.67	-0.58	0.70	0.87	1.12
200663_at	*CD63*	967	0.65	1.18	0.95	0.30	0.48	0.27	-1.19	-1.25	-1.38
200675_at	*CD81*	975	-0.77	-0.62	-1.33	1.05	1.04	1.58	0.08	-0.50	-0.53
203904_x_at	*CD82*	3732	-0.57	0.05	-1.73	1.56	-1.05	0.88	0.01	0.31	0.54
204306_s_at	*CD151*	977	1.04	0.88	0.88	0.24	-0.05	0.76	-1.39	-1.18	-1.18
205806_at	*ROM1*	6094	-0.26	1.92	0.68	0.45	-1.62	0.04	-0.83	-0.51	0.13
209114_at	*TSPAN1*	10103	-0.52	-0.57	-1.22	-0.20	-0.40	-0.61	0.37	1.93	1.21
227236_at	*TSPAN2*	10100	-0.59	-0.63	-0.84	1.55	1.34	1.07	-0.66	-0.74	-0.49
200972_at	*TSPAN3*	10099	-0.85	-1.05	-1.16	-0.18	-0.17	-0.27	1.32	1.48	0.88
209263_x_at	*TSPAN4*	7106	1.24	1.34	1.12	-0.74	-0.18	0.11	-0.91	-0.87	-1.10
209890_at	*TSPAN5*	10098	-0.35	1.55	0.71	1.27	-0.82	-0.85	-0.44	-1.31	0.23
220968_s_at	*TSPAN9*	10867	-0.32	2.15	0.31	-0.89	0.47	0.19	0.14	-1.00	-1.05
223795_at	*TSPAN10*	83882	0.35	-0.26	0.33	-0.43	-1.72	-0.08	0.00	-0.30	2.11
227610_at	*TSPAN11*	441631	2.01	-0.22	-0.96	0.73	0.44	-0.15	-0.05	-1.45	-0.37
230625_s_at	*TSPAN12*	23554	-0.43	0.90	1.40	-0.24	0.06	1.34	-1.13	-1.14	-0.77
217979_at	*TSPAN13*	27075	-1.33	-1.10	-1.14	-0.03	-0.06	0.40	1.12	1.11	1.04
223314_at	*TSPAN14*	81619	1.19	1.24	1.35	-0.60	-0.06	-0.27	-0.76	-1.17	-0.92
218693_at	*TSPAN15*	23555	-0.25	0.16	-0.05	0.62	1.42	1.30	-0.69	-0.99	-1.54
233236_at	*TSPAN16*	26526	0.99	1.18	-1.37	0.51	-1.47	-0.20	1.00	-0.13	-0.52
225235_at	*TSPAN17*	26262	0.26	0.89	0.80	0.70	0.77	0.45	-0.94	-1.61	-1.32
228819_at	*TSPAN18*	90139	-0.80	1.19	0.03	0.08	2.02	-0.32	-0.72	-0.96	-0.54
203227_s_at	*TSPAN31*	6302	-1.27	-0.54	-0.96	-0.36	-0.45	-0.20	1.47	1.24	1.06
220558_x_at	*TSPAN32*	10077	0.60	1.07	1.17	0.02	0.28	0.53	-0.81	-1.32	-1.53
225775_at	*TSPAN33*	340348	-0.11	0.00	0.29	1.18	1.05	1.12	-1.21	-1.18	-1.14
**Below Threshold**											
206625_at	*PRPH2*	5961	-0.54	-0.31	0.29	1.51	1.33	0.28	-0.05	-1.57	-0.96
209109_s_at	*TSPAN6*	7105	-0.35	-0.30	-1.51	1.39	0.83	0.82	-1.37	0.58	-0.09
202242_at	*TSPAN7*	7102	-0.53	-1.08	1.04	1.50	1.00	0.19	-1.37	-0.30	-0.45
203824_at	*TSPAN8*	7103	0.30	-0.14	1.67	-1.99	-0.53	0.84	0.17	0.01	-0.33
214624_at	*UPK1A*	11045	-0.53	0.10	0.06	0.30	-0.31	-0.39	-0.78	2.44	-0.89
210064_s_at	*UPK1B*	7348	-0.96	-0.35	-0.24	0.47	2.38	-0.36	-0.65	0.31	-0.61

Human ID			2log probe expression						Post-hoc T-Test CD1c vs CD141+
Probe Set ID	Gene Symbol	Entrez ID	Blood CD1c+R1	Blood CD1c+R2	Blood CD1c+R3	Blood CD141+ R1	Blood CD141+ R2	Blood CD141+ R3	P.value
201005_at	*CD9*	928	5.58	5.42	4.86	5.69	6.20	6.54	6.050E-02
203416_at	*CD53*	963	12.42	12.38	12.41	11.58	11.47	11.98	9.414E-03
200675_at	*CD81*	975	10.45	10.49	10.26	11.04	11.04	11.21	1.587E-03
204306_s_at	*CD151*	977	8.41	8.32	8.32	7.97	7.81	8.26	6.603E-02
203227_s_at	*TSPAN31*	6302	7.29	7.83	7.52	7.97	7.90	8.08	5.727E-02

Human ID			2log probe expression						Post-hoc T-Test CD1c vs pDCs
Probe Set ID	Gene Symbol	Entrez ID	Blood CD1c+ R1	Blood CD1c+ R2	Blood CD1c+R3	Blood pDC R1	Blood pDC R2	Blood pDC R3	P.value
201005_at	*CD9*	928	5.58	5.42	4.86	9.02	9.69	7.20	1.239E-02
203416_at	*CD53*	963	12.42	12.38	12.41	12.58	12.66	12.77	1.056E-02
200675_at	*CD81*	975	10.45	10.49	10.26	10.72	10.54	10.52	1.138E-01
204306_s_at	*CD151*	977	8.41	8.32	8.32	7.08	7.20	7.20	1.586E-05
203227_s_at	*TSPAN31*	6302	7.29	7.83	7.52	9.32	9.15	9.01	8.318E-04

Human ID			2log probe expression						Post-hoc T-Test CD141+ vs pDCs
Probe Set ID	Gene Symbol	Entrez ID	Blood CD141+ R1	Blood CD141+ R2	Blood CD141+ R3	Blood pDC R1	Blood pDC R2	Blood pDC R3	P.value
201005_at	*CD9*	928	5.69	6.20	6.54	9.02	9.69	7.20	3.347E-02
203416_at	*CD53*	963	11.58	11.47	11.98	12.58	12.66	12.77	3.882E-03
200675_at	*CD81*	975	11.04	11.04	11.21	10.72	10.54	10.52	4.483E-03
204306_s_at	*CD151*	977	7.97	7.81	8.26	7.08	7.20	7.20	3.288E-03
203227_s_at	*TSPAN31*	6302	7.97	7.90	8.08	9.32	9.15	9.01	3.395E-04

Mouse ID			2log Probe expression									Anova P.value
Probe Set ID	Gene Symbol	Entrez ID	Spleen CD4+ DC R1	Spleen CD4+ DC R2	Spleen CD4+ DC R3	Spleen CD8α+ DC R1	Spleen CD8α+ DC R2	Spleen CD8α+ DC R3	Spleen pDC R1	Spleen pDC R2	Spleen pDC R3	
10548030	*Cd9*	12527	10.01	9.86	10.28	10.33	10.48	10.84	6.84	6.72	6.48	1.009E-05
10563178	*Cd37*	12493	9.71	9.71	9.78	9.49	9.60	9.76	11.41	11.47	11.57	8.157E-06
10501063	*Cd53*	12508	11.78	11.73	12.10	11.56	11.66	11.83	12.20	12.22	12.30	1.486E-02
10367436	*Cd63*	12512	6.05	6.00	6.47	6.86	7.46	7.65	3.75	4.47	4.70	1.049E-03
10559261	*Cd81*	12520	9.59	9.37	9.65	11.18	11.35	11.46	7.39	7.33	6.81	1.009E-05
10485213	*Cd82*	12521	11.31	11.30	11.74	11.13	11.26	11.49	10.96	11.18	11.17	1.904E-01
10569198	*Cd151*	12476	8.12	8.13	7.89	8.23	8.09	7.98	7.49	7.67	7.42	6.264E-03
10445633	*Prph2*	19133	7.08	6.90	6.96	6.98	6.88	6.97	6.38	6.34	6.69	9.255E-03
10465833	*Rom1*	19881	6.65	6.49	6.51	6.28	6.47	6.48	6.59	6.97	6.77	7.132E-02
10515282	*Tspan1*	66805	5.03	5.06	4.87	5.01	5.13	5.01	5.01	5.01	5.01	5.651E-01
10494821	*Tspan2*	70747	9.34	9.11	9.16	7.50	7.86	7.80	8.65	8.65	8.89	2.738E-04
10593842	*Tspan3*	56434	9.92	9.85	10.07	10.21	10.16	10.46	9.42	9.56	9.37	1.965E-03
10558961	*Tspan4*	64540	7.09	7.04	7.00	6.98	7.07	7.22	6.84	6.96	7.00	1.828E-01
10496494	*Tspan5*	56224	6.83	6.64	6.83	6.60	6.67	6.42	6.98	7.02	6.97	9.769E-03
10598626	*Tspan7*	21912	5.29	5.26	5.42	5.47	5.53	5.29	5.22	5.27	5.08	1.203E-01
10548128	*Tspan9*	109246	8.00	7.98	8.13	7.42	7.51	7.53	6.78	6.65	6.44	9.822E-05
10383353	*Tspan10*	208634	7.61	7.17	7.27	7.37	7.39	7.70	6.95	7.33	7.10	1.904E-01
10542066	*Tspan11*	68498	7.38	7.24	7.41	7.35	7.42	7.60	7.01	7.25	7.15	6.279E-02
10543306	*Tspan12*	269831	5.13	5.13	5.13	5.13	5.13	5.13	5.10	5.06	5.13	1.509E-01
10400023	*Tspan13*	66109	12.03	11.84	12.06	11.99	12.03	12.34	12.57	12.61	12.60	6.264E-03
10419073	*Tspan14*	52588	9.09	8.92	9.34	8.71	8.75	8.44	9.56	9.41	9.51	4.776E-03
10369531	*Tspan15*	70423	6.75	6.37	6.37	6.45	6.49	6.43	6.29	6.23	5.95	1.210E-01
10405343	*Tspan17*	74257	6.55	6.54	6.54	6.54	6.76	6.63	6.43	6.50	6.10	1.265E-01
10485198	*Tspan18*	241556	6.09	6.21	5.89	6.06	6.06	6.06	6.06	6.06	6.06	9.933E-01
10373027	*Tspan31*	67125	10.28	10.08	10.31	10.03	10.12	10.00	10.93	10.96	11.01	1.093E-04
10559248	*Tspan32*	27027	5.99	5.91	5.85	6.02	5.85	5.85	5.61	5.53	5.50	3.728E-03
10536908	*Tspan33*	232670	8.61	8.69	8.62	9.32	9.34	9.51	6.50	6.56	6.51	2.874E-07
10562050	*Upk1a*	109637	6.39	6.49	6.37	6.42	6.37	6.37	6.17	6.14	6.12	1.633E-03
10439500	*Upk1b*	22268	6.29	5.96	5.93	5.81	5.98	5.98	5.98	6.13	6.05	4.561E-01
**Below Threshold**												
10606609	*Tspan6*	56496	4.65	4.61	4.57	4.77	4.69	4.69	4.69	4.69	4.83	1.153E-01
10366446	*Tspan8*	216350	4.78	5.41	4.95	4.78	4.78	4.78	4.62	4.61	4.76	1.509E-01

Mouse ID			Z-Score								
Probe Set ID	Gene Symbol	Entrez ID	Spleen CD4+ DC R1	Spleen CD4+ DC R2	Spleen CD4+ DC R3	Spleen CD8α+ DC R1	Spleen CD8α+ DC R2	Spleen CD8α+ DC R3	Spleen pDC R1	Spleen pDC R2	Spleen pDC R3
10548030	*Cd9*	12527	0.50	0.42	0.65	0.68	0.76	0.95	-1.23	-1.29	-1.43
10563178	*Cd37*	12493	-0.63	-0.62	-0.54	-0.87	-0.74	-0.57	1.24	1.32	1.42
10501063	*Cd53*	12508	-0.56	-0.73	0.61	-1.33	-0.99	-0.37	0.98	1.05	1.35
10367436	*Cd63*	12512	0.08	0.05	0.39	0.68	1.12	1.26	-1.60	-1.07	-0.91
10559261	*Cd81*	12520	0.13	0.01	0.16	1.01	1.11	1.16	-1.08	-1.11	-1.40
10485213	*Cd82*	12521	0.13	0.07	2.04	-0.69	-0.12	0.93	-1.41	-0.44	-0.51
10569198	*Cd151*	12476	0.76	0.82	0.00	1.13	0.67	0.32	-1.36	-0.75	-1.58
10445633	*Prph2*	19133	1.04	0.38	0.61	0.67	0.30	0.64	-1.55	-1.70	-0.40
10465833	*Rom1*	19881	0.37	-0.46	-0.35	-1.49	-0.56	-0.50	0.06	1.96	0.97
10515282	*Tspan1*	66805	0.14	0.65	-2.17	-0.06	1.68	-0.06	-0.06	-0.06	-0.06
10494821	*Tspan2*	70747	1.17	0.84	0.91	-1.58	-1.03	-1.12	0.15	0.15	0.50
10593842	*Tspan3*	56434	0.07	-0.12	0.47	0.84	0.72	1.52	-1.26	-0.87	-1.37
10558961	*Tspan4*	64540	0.65	0.19	-0.24	-0.37	0.46	1.90	-1.75	-0.62	-0.24
10496494	*Tspan5*	56224	0.27	-0.67	0.26	-0.85	-0.52	-1.71	1.01	1.23	0.98
10598626	*Tspan7*	21912	-0.20	-0.41	0.78	1.16	1.55	-0.20	-0.66	-0.34	-1.68
10548128	*Tspan9*	109246	0.99	0.95	1.20	0.06	0.21	0.24	-0.96	-1.18	-1.50
10383353	*Tspan10*	208634	1.22	-0.64	-0.21	0.21	0.28	1.61	-1.58	0.02	-0.92
10542066	*Tspan11*	68498	0.41	-0.41	0.58	0.23	0.61	1.66	-1.76	-0.36	-0.96
10543306	*Tspan12*	269831	0.47	0.47	0.47	0.47	0.47	0.47	-0.97	-2.34	0.47
10400023	*Tspan13*	66109	-0.67	-1.28	-0.56	-0.80	-0.67	0.36	1.14	1.26	1.22
10419073	*Tspan14*	52588	0.03	-0.39	0.64	-0.94	-0.82	-1.62	1.21	0.82	1.07
10369531	*Tspan15*	70423	1.76	0.00	0.00	0.39	0.55	0.27	-0.37	-0.65	-1.95
10405343	*Tspan17*	74257	0.21	0.17	0.17	0.17	1.38	0.68	-0.46	-0.04	-2.30
10485198	*Tspan18*	241556	0.38	1.88	-2.08	-0.03	-0.03	-0.03	-0.03	-0.03	-0.03
10373027	*Tspan31*	67125	-0.31	-0.77	-0.24	-0.91	-0.70	-0.96	1.21	1.27	1.40
10559248	*Tspan32*	27027	1.02	0.59	0.33	1.18	0.33	0.33	-0.95	-1.34	-1.48
10536908	*Tspan33*	232670	0.33	0.39	0.34	0.88	0.89	1.03	-1.30	-1.26	-1.30
10562050	*Upk1a*	109637	0.55	1.28	0.40	0.78	0.40	0.40	-1.05	-1.32	-1.45
10439500	*Upk1b*	22268	2.05	-0.40	-0.61	-1.48	-0.25	-0.25	-0.25	0.89	0.32
**Below Threshold**											
10606609	*Tspan6*	56496	-0.53	-0.95	-1.53	1.10	0.03	0.03	0.03	0.03	1.80
10366446	*Tspan8*	216350	-0.21	2.42	0.52	-0.21	-0.19	-0.21	-0.89	-0.90	-0.31

MOUSE ID			2log probe expression						Post-hoc T-Test CD4+ vs CD8α
Probe Set ID	Gene Symbol	Entrez ID	Spleen CD4+ DC R1	Spleen CD4+ DC R2	Spleen CD4+ DC R3	Spleen CD8α+ DC R1	Spleen CD8α+ DC R2	Spleen CD8α+ DC R3	P.value
10548030	*Cd9*	12527	10.01	9.86	10.28	10.33	10.48	10.84	6.041E-02
10501063	*Cd53*	12508	11.78	11.73	12.10	11.56	11.66	11.83	2.558E-01
10559261	*Cd81*	12520	9.59	9.37	9.65	11.18	11.35	11.46	1.043E-04
10569198	*Cd151*	12476	8.12	8.13	7.89	8.23	8.09	7.98	6.363E-01

MOUSE ID			2log probe expression						Post-hoc T-Test CD4+ vs pDCs
Probe Set ID	Gene Symbol	Entrez ID	Spleen CD8α+ DC R1	Spleen CD8α+ DC R2	Spleen CD8α+ DC R3	Spleen pDC R1	Spleen pDC R2	Spleen pDC R3	P.value
10548030	*Cd9*	12527	10.01	9.86	10.28	6.84	6.72	6.48	3.204E-05
10501063	*Cd53*	12508	11.78	11.73	12.10	12.20	12.22	12.30	3.560E-02
10559261	*Cd81*	12520	9.59	9.37	9.65	7.39	7.33	6.81	3.174E-04
10569198	*Cd151*	12476	8.12	8.13	7.89	7.49	7.67	7.42	8.484E-03

MOUSE ID			2log probe expression						Post-hoc T-Test CD8α vs pDCs
Probe Set ID	Gene Symbol	Entrez ID	Spleen CD8α+ DC R1	Spleen CD8α+ DC R2	Spleen CD8α+ DC R3	Spleen pDC R1	Spleen pDC R2	Spleen pDC R3	P.value
10548030	*Cd9*	12527	10.33	10.48	10.84	6.84	6.72	6.48	2.968E-05
10501063	*Cd53*	12508	11.56	11.66	11.83	12.20	12.22	12.30	2.597E-03
10559261	*Cd81*	12520	11.18	11.35	11.46	7.39	7.33	6.81	3.295E-05
10569198	*Cd151*	12476	8.23	8.09	7.98	7.49	7.67	7.42	4.918E-03
