# Supplementary material for: Differential expression of tetraspanin superfamily members in dendritic cell subsets
Source: PLoS One. 2017 Sep 7;12(9):e0184317. doi: 10.1371/journal.pone.0184317 (PMC5589240; doi:10.1371/journal.pone.0184317)
Supplement: S2 Table — (PDF) [file pone.0184317.s002.pdf]

Human Protein	2log corrected MFI									Anova p. value
**Genes**	**CD1c+ R1**	**CD1c+ R2**	**CD1c+ R3**	**CD141+ R1**	**CD141+ R2**	**CD141+ R3**	**pDC R1**	**pDC R2**	**pDC R3**	**p. value**
CD9	9.60	10.00	10.28	9.90	10.36	10.06	9.28	9.44	9.75	1.293E-01
CD37	11.23	10.86	12.54	11.32	11.03	12.36	11.11	10.86	12.35	9.760E-01
CD53	13.86	14.30	14.64	13.41	13.91	14.23	14.21	14.61	14.84	2.090E-01
CD81	12.47	12.53	12.81	13.40	13.73	13.76	9.88	9.89	10.19	6.347E-06
CD82	9.39	8.37	9.00	9.43	8.32	8.67	7.19	7.30	7.41	1.762E-02
CD151	11.02	10.64	10.58	10.71	10.41	10.26	9.17	8.77	9.19	8.198E-04
Tspan31	12.10	11.81	12.19	12.03	11.73	12.18	12.13	11.82	12.12	9.760E-01

Human Protein	2log corrected MFI						Post-hoc T-Test CD1c vs CD141+
**Genes**	**CD1c+ R1**	**CD1c+ R2**	**CD1c+ R3**	**CD141+ R1**	**CD141+ R2**	**CD141+ R3**	**p. value**
CD81	12.47	12.53	12.81	13.40	13.73	13.76	2.695E-03
CD82	9.39	8.37	9.00	9.43	8.32	8.67	8.093E-01
CD151	11.02	10.64	10.58	10.71	10.41	10.26	2.058E-01

Human Protein	2log corrected MFI						Post-hoc T-Test CD1c vs pDCs
**Genes**	**CD1c+ R1**	**CD1c+ R2**	**CD1c+ R3**	**pDC R1**	**pDC R2**	**pDC R3**	**p. value**
CD81	12.47	12.53	12.81	9.88	9.89	10.19	5.744E-05
CD82	9.39	8.37	9.00	7.19	7.30	7.41	6.006E-03
CD151	11.02	10.64	10.58	9.17	8.77	9.19	9.023E-04

Human Protein	2log corrected MFI						Post-hoc T-Test CD141+ vs pDCs
**Genes**	**CD141+ R1**	**CD141+ R2**	**CD141+ R3**	**pDC R1**	**pDC R2**	**pDC R3**	**p. value**
CD81	13.40	13.73	13.76	9.88	9.89	10.19	1.850E-05
CD82	9.43	8.32	8.67	7.19	7.30	7.41	1.063E-02
CD151	10.71	10.41	10.26	9.17	8.77	9.19	1.686E-03

Mouse Protein	2log corrected MFI									Anova p. value
Genes	Spleen CD4+ DC R1	Spleen CD4+ DC R2	Spleen CD4+ DC R3	Spleen CD8α+ DC R1	Spleen CD8α+ DC R2	Spleen CD8α+ DC R3	Spleen pDC R1	Spleen pDC R2	Spleen pDC R3	p. value
Cd9	7.65	7.50	7.62	9.14	8.75	9.02	1.26	0.00	3.29	7.049E-04
Cd53	5.76	5.53	5.54	5.57	5.65	5.53	5.41	5.26	5.22	1.620E-02
Cd81	7.70	7.57	7.47	9.62	9.70	9.48	4.73	5.99	6.64	1.082E-03
Cd151	5.24	5.46	5.20	5.83	5.87	5.89	5.41	5.67	5.80	1.065E-02

Mouse Protein	2log corrected MFI						Post-hoc T-Test CD4+ vs CD8α
Genes	Spleen CD4+ DC R1	Spleen CD4+ DC R2	Spleen CD4+ DC R3	Spleen CD8α+ DC R1	Spleen CD8α+ DC R2	Spleen CD8α+ DC R3	p. value
Cd9	7.65	7.50	7.62	9.14	8.75	9.02	3.62E-04
Cd53	5.76	5.53	5.54	5.57	5.65	5.53	7.90E-01
Cd81	7.70	7.57	7.47	9.62	9.70	9.48	2.67E-05
Cd151	5.24	5.46	5.20	5.83	5.87	5.89	2.53E-03

Mouse Protein	2log corrected MFI						Post-hoc T-Test CD4+ vs pDCs
Genes	Spleen CD4+ DC R1	Spleen CD4+ DC R2	Spleen CD4+ DC R3	Spleen pDC R1	Spleen pDC R2	Spleen pDC R3	p. value
Cd9	7.65	7.50	7.62	1.26	0.00	3.29	3.20E-03
Cd53	5.76	5.53	5.54	5.41	5.26	5.22	2.97E-02
Cd81	7.70	7.57	7.47	4.73	5.99	6.64	3.40E-02
Cd151	5.24	5.46	5.20	5.41	5.67	5.80	8.24E-02

Mouse Protein	2log corrected MFI						Post-hoc T-Test CD8α vs pDCs
Genes	Spleen CD8α+ DC R1	Spleen CD8α+ DC R2	Spleen CD8α+ DC R3	Spleen pDC R1	Spleen pDC R2	Spleen pDC R3	p. value
Cd9	9.14	8.75	9.02	1.26	0.00	3.29	1.52E-03
Cd53	5.57	5.65	5.53	5.41	5.26	5.22	1.24E-02
Cd81	9.62	9.70	9.48	4.73	5.99	6.64	2.52E-03
Cd151	5.83	5.87	5.89	5.41	5.67	5.80	1.11E-01
