# Supplementary material for: Differential expression of tetraspanin superfamily members in dendritic cell subsets
Source: PLoS One. 2017 Sep 7;12(9):e0184317. doi: 10.1371/journal.pone.0184317 (PMC5589240; doi:10.1371/journal.pone.0184317)
Supplement: S3 Table — (DOCX) [file pone.0184317.s003.docx]

**Table S3. Antibodies.**

| **Anti-human antibodies** | | |
| --- | --- | --- |
| CD9 | M-L13 | BD, 555370 |
| CD37 | WR17 | Home made |
| CD53 | Mem53 | Serotec, MCA7236 |
| CD81 | JS-81 | BD, 555675 |
| CD82 | BL-2 | Novus Bio, NB600-1283 |
| CD151 | 11G5a | Serotec, MCA1856 |
| Tspan31 | Rabbit polyclonal | Abgent, AP8587c |
| lineage-FITC | Lin 1 cocktail | BD, 340546 |
| BDCA4-PerCP-Cy5.5 | 12C2 | BioLegend, 354509 |
| BDCA3-PE | - | Miltenyi |
| BDCA3-APC | AD5-14H12 | Miltenyi, 130-090-907 |
| BDCA1-BV421 | L161 | BioLegend, 331526 |
| MHC class II-BV510 | G46-6 | BD, 563083 |
| **Anti-mouse antibodies:** | | |
| CD9 | KMC8 | eBioscience, 14-0091-82 |
| CD53-FITC | OX-79 | BioLegend, 124705 |
| CD81 | Eat-2 | BioLegend, 104902 |
| CD151 | 455807 | R&D, MAB4609 |
| CD11c-PerCP | N418 | BioLegend, 117326 |
| B220-PE | RA3-682 | BD, 553089 |
| CD11b-PE | M1/70 | BioLegend, 101208 |
| CD8α-FITC | 53-6.7 | BD, 553031 |
| CD8α-APC | 53-6.7 | BioLegend, 100712 |
| CD4-FITC | RM4-5 | BioLegend, 100510 |
| CD4-APC | RM4-5 | BioLegend, 100516 |
| Siglec-H-alexa647 | 551 | Biolegend, 129608 |
| **Isotype control antibodies:** | | |
| Mouse IgG1 | MOPC-21 | BioLegend, 400102 |
| Mouse IgG2a | MOPC-173 | BioLegend, 400202 |
| Rabbit IgG | polyclonal | Jackson IE, 011-000-003 |
| Rat IgG2a | eBR2a | eBioscience, 14-4321-85 |
| Armenian Hamster IgG | eBio299Arm | eBioscience, 14-488-81 |
| **Secondary antibodies:** | | |
| anti-mouse-PE |  |  |
| Goat anti-rabbit-alexa647 | polyclonal | Life Technologies, A-21245 |
| Goat-anti-rat-alexa647 | polyclonal | Life Technologies, A-21247 |
| Goat anti-hamster (Armenian)-biotin | Poly4055 | BioLegend, 405501 |
| **Other:** | | |
| Streptavidin-alexa647 |  | Life Technologies, S-32357 |
| Fixable Viability dye eFluor780 |  | eBioscience, 65-0865-14 |
