# Supplementary figures and images for: Differential expression of tetraspanin superfamily members in dendritic cell subsets
Source: PLoS One. 2017 Sep 7;12(9):e0184317. doi: 10.1371/journal.pone.0184317 (PMC5589240; doi:10.1371/journal.pone.0184317)

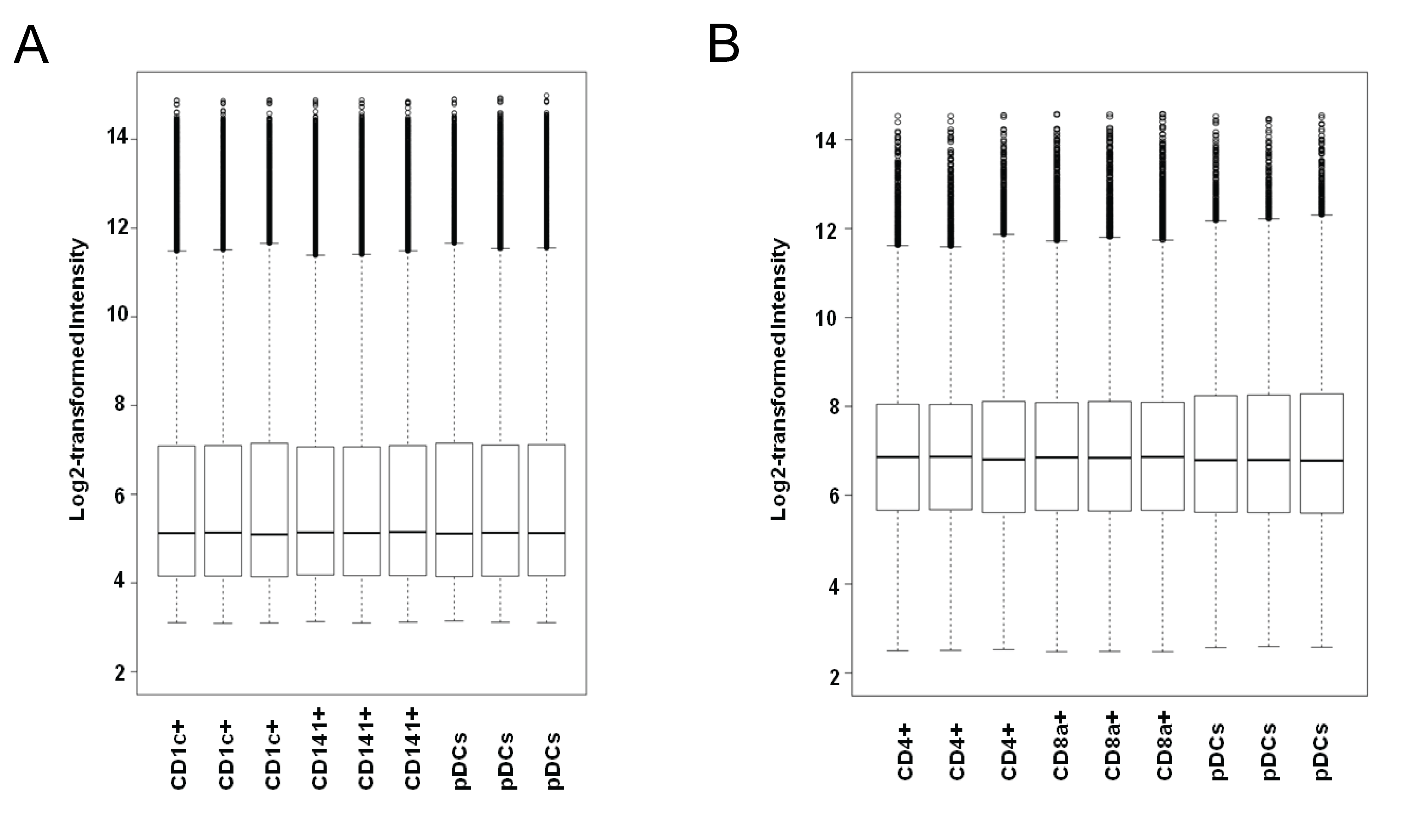

Supplement: S1 Fig — Box plots of normalized probe intensity distributions. Complete data set of expression values of human blood DC subsets (A) and murine spleen DC subsets (B) were normalized using the RMA normalization function and 2log transformation. (TIF) [file pone.0184317.s004.tif]

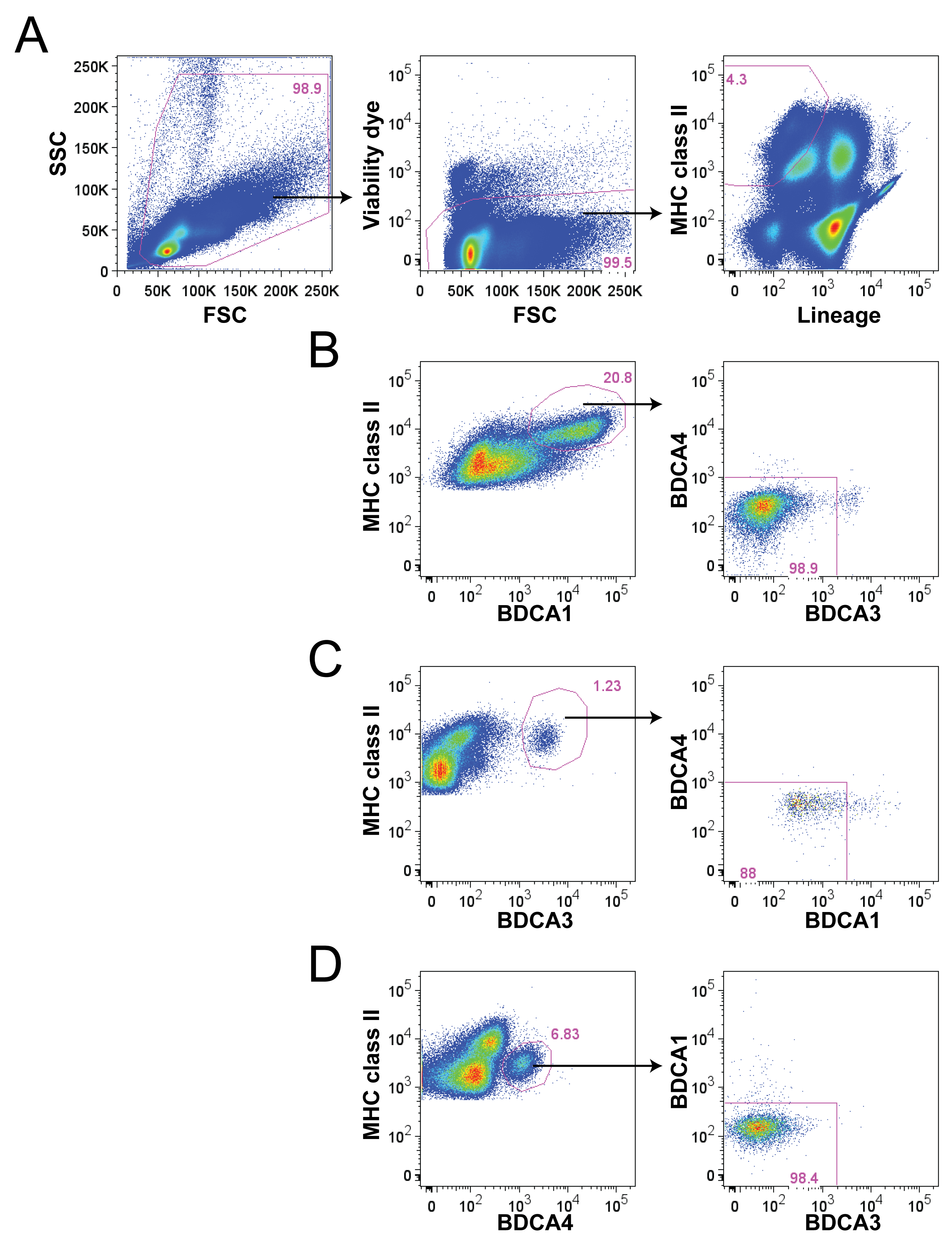

Supplement: S2 Fig — PBMCs were stained with specific antibodies and analyzed by flow cytometry. (A) Cell debris (left) and cells positive for dead cell marker were excluded (middle). DCs positive for MHC class II and negative for lineage markers CD3, CD14, CD16, CD19, CD20 and CD56 were selected (right). (B) CD1c+ DCs were gated on MHC class II and BDCA1 positivity (left). Cells positive for BDCA3 and BDCA4 were excluded (right) from tetraspanin expression analysis. (C) CD141+ DCs were gated on MHC class II and BDCA3 positivity (left). Cells positive for BDCA1 and BDCA4 were excluded (right) from tetraspanin expression analysis. (D) pDCs were gated on MHC class II and BDCA4 positivity (left). Cells positive for BDCA1 and BDCA3 were excluded (right) from tetraspanin expression analysis. (TIF) [file pone.0184317.s005.tif]

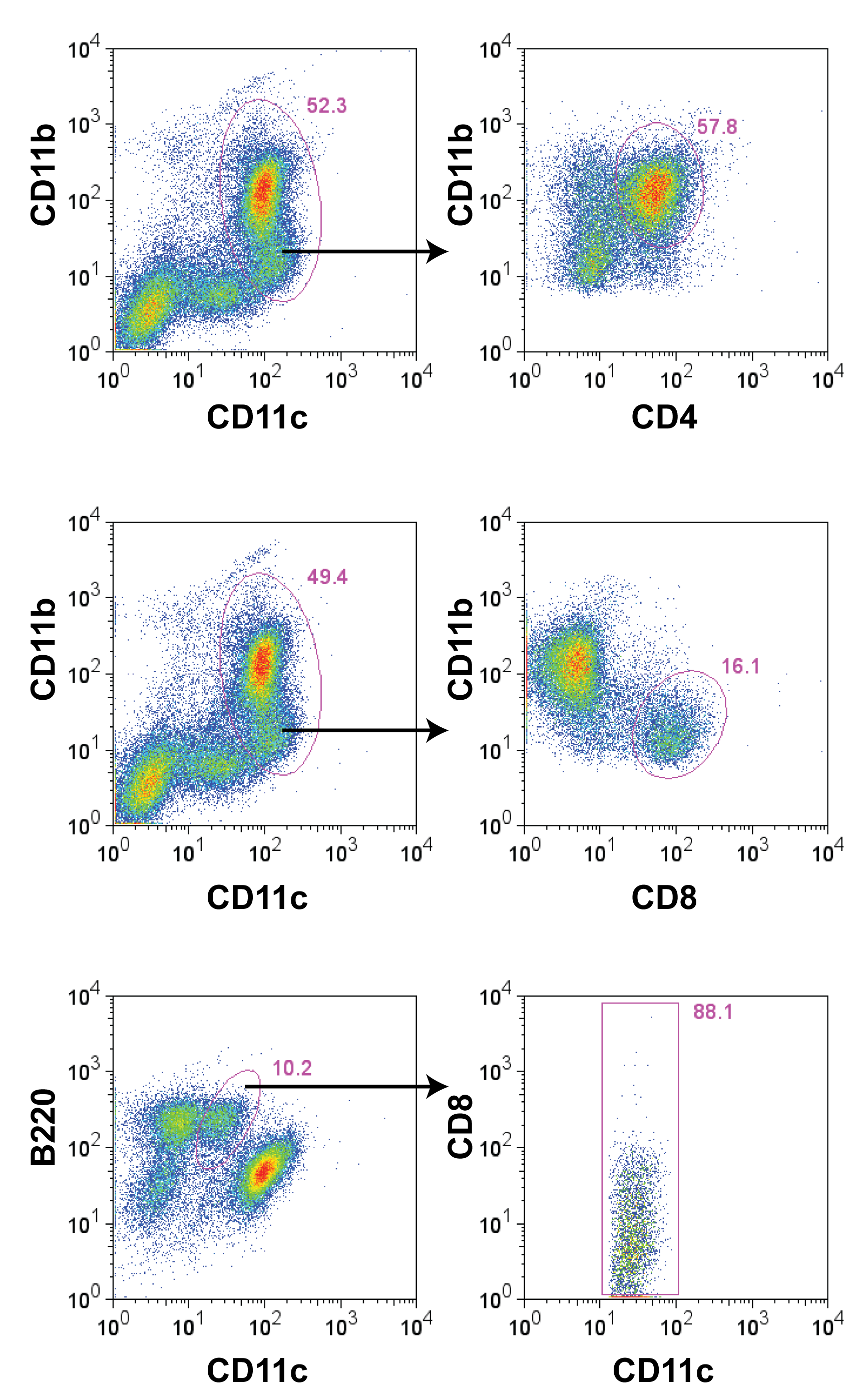

Supplement: S3 Fig — Splenic cells were enriched for DCs, stained with specific antibodies and analyzed by flow cytometry. Dead cells were excluded based on forward and side scatter characteristics. Upper: CD11c+ cells were gated on CD11b+ CD4+ to determine tetraspanin expression on CD4+ DCs. Middle: CD11c+ CD11b- CD8α+ cells were selected for tetraspanin expression analyses. Lower, pDCs: B220+ CD11cint. cells were gated on CD8α+ for tetraspanin expression analyses. (TIF) [file pone.0184317.s006.tif]
